# Successful endoscopic sphincterotomy using a novel rotatable sphincterotome in a patient with Roux-en-Y gastrectomy

**DOI:** 10.1055/a-2463-3966

**Published:** 2024-11-26

**Authors:** Yuki Tanisaka, Shomei Ryozawa, Masafumi Mizuide, Akashi Fujita, Ryuhei Jinushi, Ryuichi Watanabe, Ryo Sato

**Affiliations:** 1 183786 Department of Gastroenterology, Saitama Medical University International Medical Center, Hidaka, Japan

Balloon enteroscopy-assisted endoscopic retrograde cholangiopancreatography (ERCP) is reported to be useful for patients with Roux-en-Y gastrectomy 1 2 3 . However, performing endoscopic sphincterotomy (EST) is considered challenging because the papilla appears inverted and its position is often tangential, which makes visualizing the correct direction for the incision (5 o’clock direction) difficult. Although there are several sphincterotomes facilitating EST in such cases, they do not always ensure the correct direction for EST 4 5 .

A novel rotatable sphincterotome (ENGETSU; Kaneka Corp., Osaka, Japan) was launched to overcome this limitation ( Fig. 1 ). The blade of the sphincterotome can be rotated by the endoscopist by turning a handle, which facilitates easier and safer EST ( Fig. 2 ). We report a case of successful EST using this novel rotatable sphincterotome in a patient with Roux-en-Y gastrectomy.

**Fig. 1:**
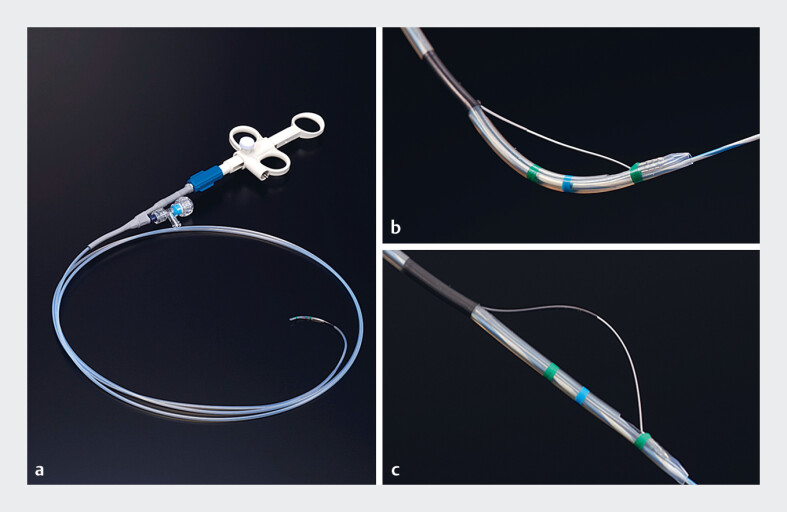
The novel rotatable sphincterotome (ENGETSU; Kaneka Corp., Osaka, Japan). **a** Product overview. **b** The blade can be stretched. **c** The blade can also be loosened. Source: Kaneka Corp.

**Fig. 2:**
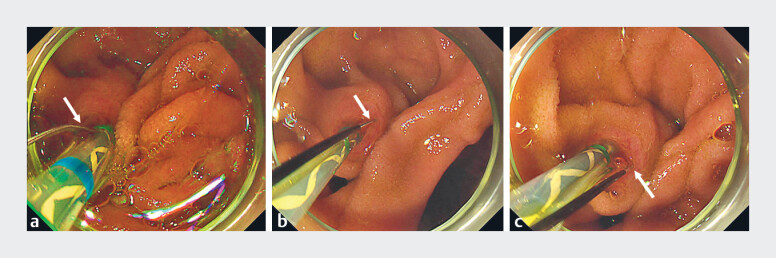
Endoscopic findings. **a** The blade of the sphincterotome was initially facing the 1 o’clock direction (red arrow). **b** The blade of the sphincterotome was rotated to the 3 o’clock direction (red arrow) by turning the handle. **c** The blade of the sphincterotome was rotated to the 5 o’clock direction (red arrow) by turning the handle.

A 71-year-old woman presenting with cholangitis due to suspected debris in the common bile duct (CBD) was referred to our facility. She underwent Roux-en-Y gastrectomy owing to gastric cancer. ERCP was performed using a short-type single-balloon enteroscope (SIF-H290; Olympus, Tokyo, Japan) with a working length of 152 cm and a working channel diameter of 3.2 mm 2 ( Video 1 ). After reaching the papilla and achieving selective biliary cannulation, EST was attempted with the novel rotatable sphincterotome. Although the blade of the sphincterotome was not initially positioned in the 5 o’clock direction, we were able to adjust it to face 5 o’clock by turning its handle ( Fig. 3 **a, b** ). EST was performed effectively and safely, with the blade correctly oriented for the incision ( Fig. 3 **c** ). Finally, the CBD was cleared using a basket catheter ( Fig. 3 **d** ).

Successful endoscopic sphincterotomy using a novel rotatable sphincterotome in a patient with Roux-en-Y gastrectomy.Video 1

**Fig. 3:**
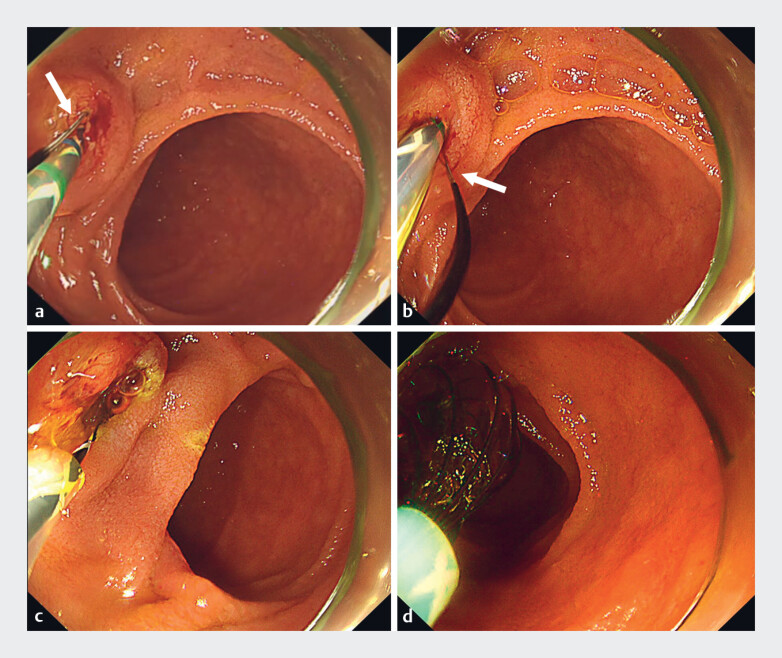
Endoscopic findings. **a** The blade of the sphincterotome was not initially positioned in the 5 o’clock direction (red arrow). **b** The blade was adjusted to face 5 o’clock (red arrow) by turning the handle. **c** Endoscopic sphincterotomy was safely performed. **d** The common bile duct was cleared using a basket catheter.

Compared with a previously reported sphincterotome 4 , this novel rotatable sphincterotome allows for greater rotation as required by the endoscopist. It can improve the success rate of EST in patients with Roux-en-Y gastrectomy.

Endoscopy_UCTN_Code_TTT_1AR_2AC

## Notes

Endoscopy E-Videos https://eref.thieme.de/e-videos
*E-Videos* is an open access online section of the journal *Endoscopy* , reporting on interesting cases and new techniques in gastroenterological endoscopy. All papers include a high-quality video and are published with a Creative Commons CC-BY license. Endoscopy E-Videos qualify for HINARI discounts and waivers and eligibility is automatically checked during the submission process. We grant 100% waivers to articles whose corresponding authors are based in Group A countries and 50% waivers to those who are based in Group B countries as classified by Research4Life (see: https://www.research4life.org/access/eligibility/ ). This section has its own submission website at https://mc.manuscriptcentral.com/e-videos .
